# Reductions in extracellular vesicle-associated microRNA-126 levels in coronary blood after acute myocardial infarction: A retrospective study

**DOI:** 10.3389/fcvm.2022.1046839

**Published:** 2022-11-29

**Authors:** Yujuan Yuan, Yiping Ma, Zulipiya Aili, Muyesai Nijiati

**Affiliations:** ^1^Department of Cardiology, People’s Hospital of Xinjiang Uygur Autonomous Region, Ürümqi, China; ^2^Xinjiang Emergency Center, People’s Hospital of Xinjiang Uygur Autonomous Region, Ürümqi, China

**Keywords:** acute myocardial infarction (AMI), microparticles (MPs), miR-126, coronary blood, TIMI score, thrombosis

## Abstract

**Background:**

Acute Myocardial Infarction (AMI) is a kind of cardiovascular disease with high mortality and incidence. Extracellular vesicles (EVs) and microRNA-126 (miR-126) are known to play important role in the development and prognosis of several cardiovascular diseases. Therefore, this study aimed to investigate the changes in Extracellular vesicle (EV)-associated miR-126 levels in the coronary blood of patients with AMI to explore the relationship between miR-126 levels and AMI.

**Materials and methods:**

We analyzed EV-associated miR-126 in the coronary blood of patients with AMI and stable coronary artery disease (SCAD) using quantitative reverse transcription polymerase chain reaction (qRT-PCR).

**Results:**

We tested the coronary blood of 20 patients with AMI and 20 with SCAD. The mean age of the patients was 58.8 ± 10.3 years and 32 (80%) were men. We observed that the EV-associated miR-126 levels were lower in patients with AMI [median = 0.13; interquartile range (IQR): 0.08–0.22] than in patients with SCAD (median = 0.37; IQR: 0.26–0.48) (*P* < 0.001). In addition, the levels of miR-126 were negatively associated with the Thrombolysis in Myocardial Infarction (TIMI) score (*r* = −0.66, *P* = 0.001).

**Conclusion:**

Reduction of EV-associated miR-126 levels in the coronary blood of patients with AMI may be involved in acute coronary thrombosis events.

## Introduction

Coronary heart disease is a leading cause of global morbidity and mortality and is triggered by coronary atherosclerosis, a condition that causes occlusion and stenosis of the coronary arteries (1–5). In 2020, approximately 19 million deaths are attributed to cardiovascular diseases worldwide. Between 2010 and 2020, there was an 18.7% increase in the incidence of deaths due to cardiovascular diseases (4). Each year, it is estimated that > 7 million people worldwide are diagnosed with acute coronary syndrome (ACS) (6). Acute myocardial infarction (AMI) is a serious coronary condition and is diagnosed in approximately 700,000 people worldwide (7).

Extracellular vesicles (EVs) are small and lipid membrane particles secreted by cell activation or apoptosis into the extracellular environment. According to size and biogenesis, EVs are mainly divided into three categories: exosomes, microvesicles, and apoptotic bodies (8, 9). Several studies demonstrated that EVs could be involved in the pathophysiology of many diseases, including cardiovascular, neurological, and autoimmune diseases (10–13). The circulating EVs are recognized by and bind to specific surface proteins on target cells. This binding releases proteins, mRNAs, microRNAs (miRNAs), and other substances into the target cells, which activate various signaling pathways to regulate the functional states of the target cells (11, 14).

MiRNAs are 21–23 nucleotides long, highly conserved, non-coding small RNA molecules. Due to their effects on gene expression and association with disease conditions, they are being intensely researched as potential biomarkers (15–17). Since miRNAs are endogenous post-translational regulatory genes, they regulate gene expression by binding to mRNAs and promoting their degradation or translation. The miRNAs in blood have attracted extensive attention as biomarkers of cardiovascular disease (18, 19). Recently, several studies have shown that EVs and miRNAs play important roles in patients with coronary artery disease (CAD) as they are involved in endothelial dysfunction (20–22). The endothelium-associated miR-126 is one of the most abundant miRNAs in endothelial cells and is associated with endothelial permeability and apoptosis (23, 24). Clinical studies have demonstrated that miR-126 levels serve as a novel biomarker for CAD, which is associated with a decrease in miRNA-126 levels in blood (25–27). Jansen et al. also found miR-126 levels in circulating microvesicles were significantly associated with a decreased risk of cardiovascular events in patients with stable coronary artery disease (SCAD) (28). However, these studies have focused only on the overall circulating levels of miR-126 and have not investigated their levels in EVs from coronary blood, especially in patients with AMI. Hence, this study investigated the levels of EV-associated miR-126 in the coronary blood of patients with AMI. In addition, it also analyzed the correlation between miR-126 levels and Thrombolysis in Myocardial Infarction (TIMI) scores.

## Materials and methods

### Study population

In all, we recruited 40 patients who were admitted to the People’s Hospital of Xinjiang Uygur Autonomous Region between 1st January and 31st December, 2019. Of these, 20 patients had AMI and 20 had SCAD. All patients who met the inclusion and exclusion criteria and signed the informed consent were included in the study. The inclusion and exclusion criteria used in this study were as mentioned in Yuan et al. (29).

Briefly, the inclusion criteria were:

(a) For patients with AMI: Patients having elevated cardiac biomarker levels that exceeded the 99th percentile of the upper limit of the reference range and exhibited at least one of the following conditions: (1) Symptoms of myocardial ischemia, (2) new ischemic electrocardiogram (ECG) changes, (3) presence of a pathological Q-wave in the ECG, (4) imaging evidence of new loss of viable myocardium or new regional wall motion abnormality in a pattern consistent with an ischemic etiology, and (5) identification of a coronary thrombus by angiography or autopsy.
(b) For patients with SCAD: (1) Patients having a clinical syndrome of transient ischemia and hypoxia caused by increased myocardial load based on fixed and severe coronary artery stenosis. (2) Patients undergoing coronary angiography for the diagnosis of atherosclerotic heart disease and stent implantation were included (refer to guidelines for the Diagnosis and Treatment of Stable Coronary Artery Disease, Chinese Journal of Cardiovascular Diseases, 2018).

The exclusion criteria were:

(1) Serious liver or kidney dysfunction, (2) cancer or other debilitating diseases, (3) diseases of the hematopoietic system, (4) uncontrolled infection, (5) infarction in another location of the body, such as cerebral infarction or pulmonary embolism, and (6) coronary artery spasm.

### Clinical data collection

We collected the following relevant clinical data from the electronic medical record system: (1) Details of age, sex, body weight and height, smoking or drinking status, heart rate, history of diabetes mellitus and hypertension. (2) Levels of creatinine (Cr), triglycerides (TG), total cholesterol (TC), low-density lipoprotein-cholesterol (LDL-C), and high-density lipoprotein-cholesterol (HDL-C) in blood. (3) Levels of left ventricular ejection fraction (LVEF). (4) History of use of statins and antiplatelet agents. All data were collected from fasting blood samples after admission. Body mass index (BMI) = weight (kg)/height^2 (m^2^).

### Sample collection

Coronary blood extraction: During percutaneous coronary intervention (PCI) surgery, doctors entered the coronary artery through the subject’s radial artery. Once the guide wire reached the lesion site and the balloon entered the plaque, it was dilated to accommodate the coronary arteries. After the rapid release of the balloon, 5 ml of coronary blood at the front end was discarded, following which, 10 ml of coronary blood was extracted before the balloon was removed from the guide wire. The coronary blood samples were centrifuged at 3,500 *g* for 15 min at 4°C. The supernatant obtained was stored in EDTA EP tubes at −80°C.

### Isolation of extracellular vesicles

A portion of the coronary blood sample dissolved at room temperature was removed from the EDTA tubes. These samples were centrifuged at 2,700 *g* for 15 min at 4°C and the supernatants were transferred to new EP tubes and centrifuged again at 2,700 *g* for 5 min at 4°C. After two more rounds of centrifugation and supernatant collection, the samples were centrifuged at 20,000 *g* for 20 min at 4°C and the supernatant was gently removed.

### Analysis of microRNA-126 levels in extracellular vesicles isolated from blood samples of patients with acute myocardial infarction and stable coronary artery disease

We extracted total RNA from EVs using TRIzol reagent (TaKaRa, 9108, Japan) and determined their concentrations and purities using NanoDrop 2000 (Thermo Fisher, ECS000282, USA). The RNA samples were reverse transcribed into cDNA by using the Bugle-Loop™ RT reagent Kit (Ribobio China). The levels of miR-126 were measured using the 7300 Real-Time PCR system (Applied Biosystems, StepOnePlus, USA) and SYBR Green Master Mix (Takara, RR820A, Japan). The reaction conditions were set as follows: initial denaturation at 95°C for 10 min followed by 40 cycles of denaturation at 95°C for 2s, annealing at 60°C for 20 s, and extension at 70°C for 10 s. The endogenous control was from U6 (Ribobio China) and the 2^–ΔΔ*Ct*^ method was used to calculate the miR-126 expression levels.

### Statistical analysis

We used R v. 4.2.1 for data analyses. Continuous variables were tested for normality with the Kolmogorov–Smirnov test. Normally distributed data are expressed as mean ± standard deviation and were compared using the Student’s *t*-test. Non-normal data are expressed as median ± interquartile range (IQR) and were compared using the Mann–Whitney *U* test. The categorical variables are presented as frequencies and proportions and were compared using the Chi-square test. Pearson’s correlation coefficient was calculated to determine the relationship between the levels of miR-126 and TIMI scores. A *P*-value of < 0.05 was considered statistically significant.

## Results

### Clinical characteristics of acute myocardial infarction and stable coronary artery disease groups

In all, 40 patients (20 with AMI and 20 with SCAD) were recruited in this study. The mean age of the patients was 58.8 ± 10.3 years and 32 (80%) were men. The baseline characteristics of the patients are presented in Table 1. No significant differences were noted between the two groups in terms of age, sex, body mass index (BMI), history of hypertension and diabetes mellitus, and smoking habits. Similarly, we observed no significant differences in blood levels of TC, TG, HDL-C, LDL-C, and Cr between the two groups. In addition, there were no differences in LVEF or use of statins and antiplatelet agents between the two groups.

**TABLE 1:** Baseline clinical characteristics of patients with AMI and SCAD.

Variables	AMI (*n* = 20)	SCAD (*n* = 20)	*P-value*
Age (years)	58.6 ± 8.57	59.0 ± 12.1	0.916
Sex			0.695
Male (*n*, %)	15 (75.0%)	17 (85.0%)	
Female (*n*, %)	5 (25.0%)	3 (15.0%)	
Smoking (*n*, %)	9 (45.0%)	12 (60.0%)	0.527
BMI (Kg/m^2^)	26.6 ± 2.69	26.1 ± 3.45	0.563
SBP (mmHg)	124 ± 18.1	125 ± 20.2	0.961
DBP (mmHg)	74.0 ± 11.1	76.8 ± 13.8	0.469
Heart rate (beats/min)	75.0 ± 11.4	82.8 ± 11.2	0.034
Cr (μmol/l)	71.8 ± 12.9	70.9 ± 20.5	0.867
TG (mmol/L)	1.10 (0.98, 1.42)	1.35 (0.91, 2.00)	0.818
TC (mmol/L)	3.86 ± 1.01	4.05 ± 0.92	0.536
HDL-cholesterol (mmol/L)	1.00 ± 0.25	1.03 ± 0.30	0.738
LDL-cholesterol (mmol/L)	2.18 ± 0.96	2.45 ± 0.94	0.366
LVEF (%)	59.0 ± 5.66	56.2 ± 6.74	0.156
Hypertension (*n*, %)	12 (60.0%)	9 (45.0%)	0.527
Diabetes mellitus (*n*, %)	4 (20.0%)	5 (25.0%)	1.000
Statins (*n*, %)	6 (30.0%)	4 (20.0%)	0.715
Antiplatelet agents (*n*, %)	5 (25.0%)	5 (25.0%)	1.000

BMI, body mass index; SBP, systolic blood pressure; DBP, diastolic blood pressure; AMI, acute myocardial infarction; SCAD, stable coronary artery disease; Cr, creatinine; TG, triglycerides; TC, total cholesterol; LDL-cholesterol, Low-density lipoprotein-cholesterol; HDL-cholesterol, High-density lipoprotein-cholesterol; LVEF, left ventricular ejection fraction.

### Levels of microRNA-126 in extracellular vesicles isolated from coronary blood

**FIGURE 1:**
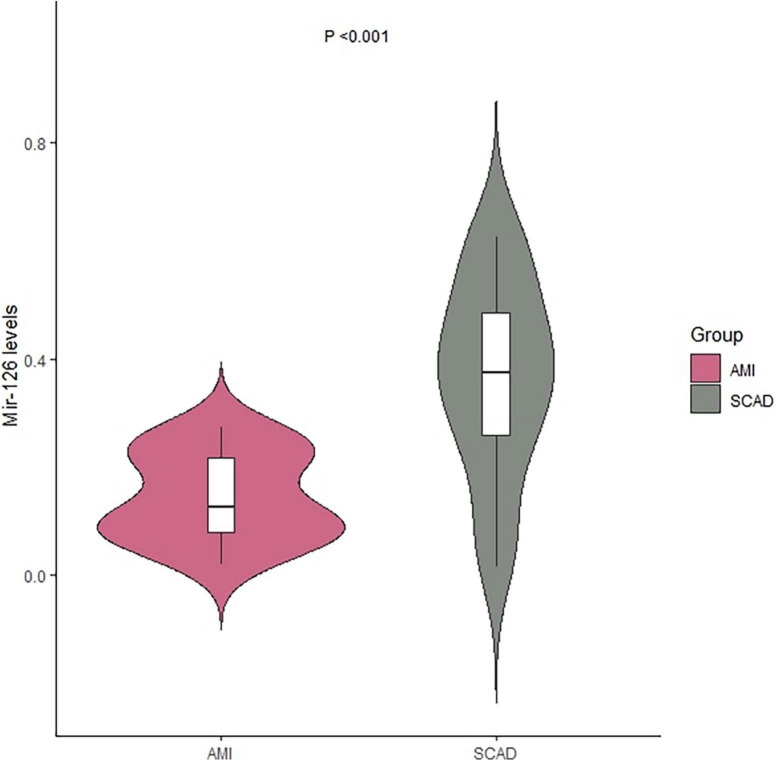
Levels of extracellular vesicle-associated miR-126 in coronary blood of patients with AMI and SCAD. AMI, acute myocardial infarction; SCAD, stable coronary artery disease.

The miR-126 levels in EVs from coronary blood were significantly lower in patients with AMI (median = 0.13; IQR: 0.08–0.22) than those with SCAD (median = 0.37; IQR: 0.26–0.48) (*P* < 0.001) (Table 2 and Figure 1).

**TABLE 2:** Levels of extracellular vesicle-associated miR-126 in coronary blood of patients with AMI and SCAD.

	AMI	SCAD	*P-value*
Mir-126	0.13 (0.08, 0.22)	0.37 (0.26, 0.48)	< 0.001

AMI, acute myocardial infarction; SCAD, stable coronary artery disease.

### Association between microRNA-126 levels and Thrombolysis in Myocardial Infarction score

**FIGURE 2:**
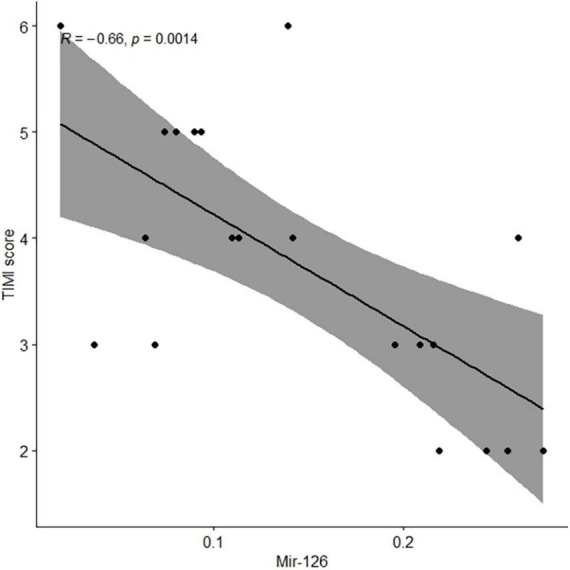
Levels of extracellular vesicle-associated miR-126 in coronary blood of patients with AMI were negatively associated with TIMI scores. AMI, acute myocardial infarction.

In clinical practice, the TIMI risk score is used to judge the prognosis of patients with the ACS and for selecting the best treatment plan for patients. We observed that the EV-associated miR-126 levels in coronary blood were significantly negatively associated with TIMI scores in patients with AMI (*r* = −0.66, *P* = 0.001) (Figure 2).

## Discussion

Acute Myocardial Infarction is typically caused due to obstructions in the coronary artery, which prevents blood from flowing to the myocardium. Research has shown that miRNAs are packed into EVs to protect them from degradation by nucleotidases. Therefore, miRNAs in the EVs, which are released during cell death, serve as intercellular signal transduction agents (30).

Studies have shown that miRNAs play important roles in the occurrence and development of several diseases (31, 32). The changes in miRNA levels associated with cardiovascular diseases have attracted attention since they have significant diagnostic values (33, 34). Chen et al. showed that a decrease in miRNA-126-3p level plays a role in promoting the development of lung cancer; therefore, it could be used as a biomarker for the early diagnosis and prognosis of lung cancer (35). Wang et al. demonstrated that miR-126 levels are significantly lower in the peripheral blood of patients with AMI than those in the control group (26). In addition, miR-126 may also be involved in regulating vascular integrity and angiogenesis after AMI (36).

The levels of endothelium-related miR-126-3p and miR-126-5p in EVs are associated with gene regulation in lymphocyte differentiation and activity. Additionally, they are also involved in the positive regulation of cell adhesion and the negative regulation of cell motor plexus (37). Recently, a study demonstrated that EVs rich in miR-126 are preferentially accumulated in the spleen, where they induce the localized expression of inflammatory genes and chemokine proteins and mobilize splenic neutrophils to enter peripheral blood (38). Our results show that patients with AMI have lower levels of miR-126 in EVs obtained from coronary blood than those with SCAD; however, the specific molecular mechanisms that lead to this phenomenon are still unknown and need to be explored.

Multivariate logistic regression analysis was used to select variables from TIMI trial populations for prognostic evaluation of patients and selecting the right treatment plan for them. The TIMI score predicts the occurrence of major adverse cardiac and cerebrovascular event in the next 14 days after ACS event has occurred. Our study showed that the EV-associated miR-126 levels in coronary blood samples were negatively associated with the TIMI scores in patients with AMI. This suggests that EV-associated miR-126 levels in coronary blood may indicate prognosis in AMI.

Despite the clear results obtained, the study has several limitations. (1) Since the blood samples in this study were taken from the coronary artery, the levels of EV-associated miR-126 could not be monitored repeatedly. However, since coronary blood is usually obtained from all patients during emergency PCI, it can be used to gain information on local pathological changes in coronary artery lesions. (2) Sorting experiments showed that endothelial cells were the main cellular source of EV-associated miR-126. However, we did not identify the cellular source of EVs bearing miR-126. After that, we will verify the main cellular source of EV-associated miR-126. (3) Our study has a very small sample size due to difficulties in obtaining coronary blood. We plan to expand our sample sizes in the future to ensure the robustness of our results. We also aim to gather follow-up data to investigate the relationship between levels of EV-associated miR-126 and the prognosis of patients with AMI. In addition, studies that investigate the molecular mechanisms that connect miR-126 levels in EVs from coronary blood to the occurrence of acute thrombotic events in AMI are needed. EV-associated miR-126 was studied and future studies will be focusing on the total circulating levels in coronary blood and associated to lipoproteins.

## Conclusion

A decrease in the levels of EV-associated miR-126 in the coronary blood of patients with AMI may be an indicator of acute coronary thrombosis events. However, future trials involving randomized controls and molecular biology investigations using animal models are needed to determine how levels of EV-associated miR-126 in coronary blood are related to the incidence of acute thrombosis events in AMI.

## Data availability statement

The data can be obtained by contacting the corresponding author.

## Ethics statement

The studies involving human participants were reviewed and approved by the Ethics Committee of People’s Hospital of Xinjiang Uygur Autonomous Region. The patients/participants provided their written informed consent to participate in this study.

## Author contributions

YY prepared the original manuscript, carried out the statistical analysis, and revised the submission. MN conceived the present study. YM and ZA participated in the case quality control. All authors have approved to publish the article.
